# Feasibility of Transoral Approach to Accessory Parotid Tumors

**DOI:** 10.7759/cureus.4003

**Published:** 2019-02-04

**Authors:** Suresh Mani, John Mathew, Regi Thomas, Rajiv C Michael

**Affiliations:** 1 Otolaryngology, Christian Medical College Hospital, Vellore, IND

**Keywords:** parotid gland, facial nerve, pleomorphic adenoma, transoral surgery

## Abstract

Tumors of the accessory parotid gland are very rare. Surgical removal of an accessory parotid tumor is usually accomplished by superficial parotidectomy through an external neck incision. However, this procedure inevitably results in a neck scar. In this case, we performed complete excision of a parotid tumor via an endoscopic-assisted transoral approach. Resection of such benign tumors can be achieved with less morbidity by endoscope-assisted surgery with a nerve monitoring system. The field of transoral surgery will continue to expand with technological advancements.

## Introduction

Most parotid tumors are benign and present as slow growing, painless masses in front of or below the ear lobule. Sometimes patients can present with a painless mass in the mid-cheek region, and the evaluation can be challenging given the variety of differential diagnoses like lesions from skin, lymphatic, adnexal, neurogenic, and salivary structures [1]. Nearly 60% of benign tumors of the mid-cheek region originate from the accessory parotid glands [2]. The recommended treatment is the removal of these tumors by superficial parotidectomy which encompasses facial nerve identification and en bloc removal of the superficial portion of the gland using a modified Blair’s incision or facelift incision [3]. This approach has various problems including potential injury to the facial nerve or development of a salivary fistula, cosmetic deformity, and Frey’s syndrome. Also, patients may worry about surgical scars, especially when a hypertrophic scar or keloid occurs at the site [4].

Transoral minimally invasive surgery is an emerging alternative option to the traditional open approach in select cases due to lower morbidity and good cosmetic outcome without surgical scars. This case report discusses a resection of an accessory parotid benign tumor via a transoral approach and the feasibility of such a procedure.

## Case presentation

**Figure 1:**
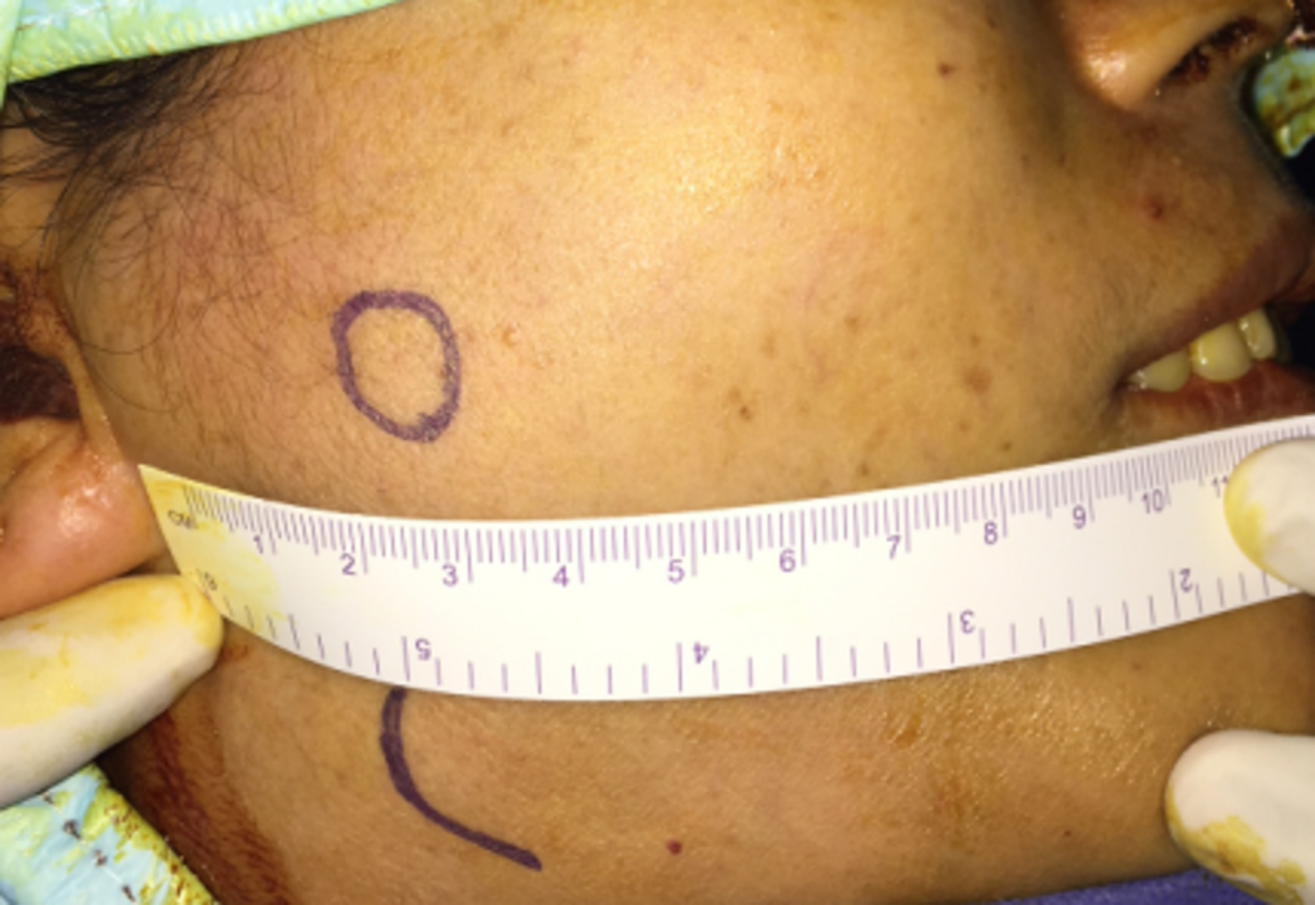
Image of 1 cm x 1 cm painless, firm mass on the patient’s right cheek area (black circle denotes the tumor)

A 43-year-old woman presented with a chief concern of painless swelling in the right cheek, which she had first noticed two years ago. On examination, we noted a 1 cm x 1 cm painless, firm mass in the patient’s right cheek area (Figure 1).

**Figure 2:**
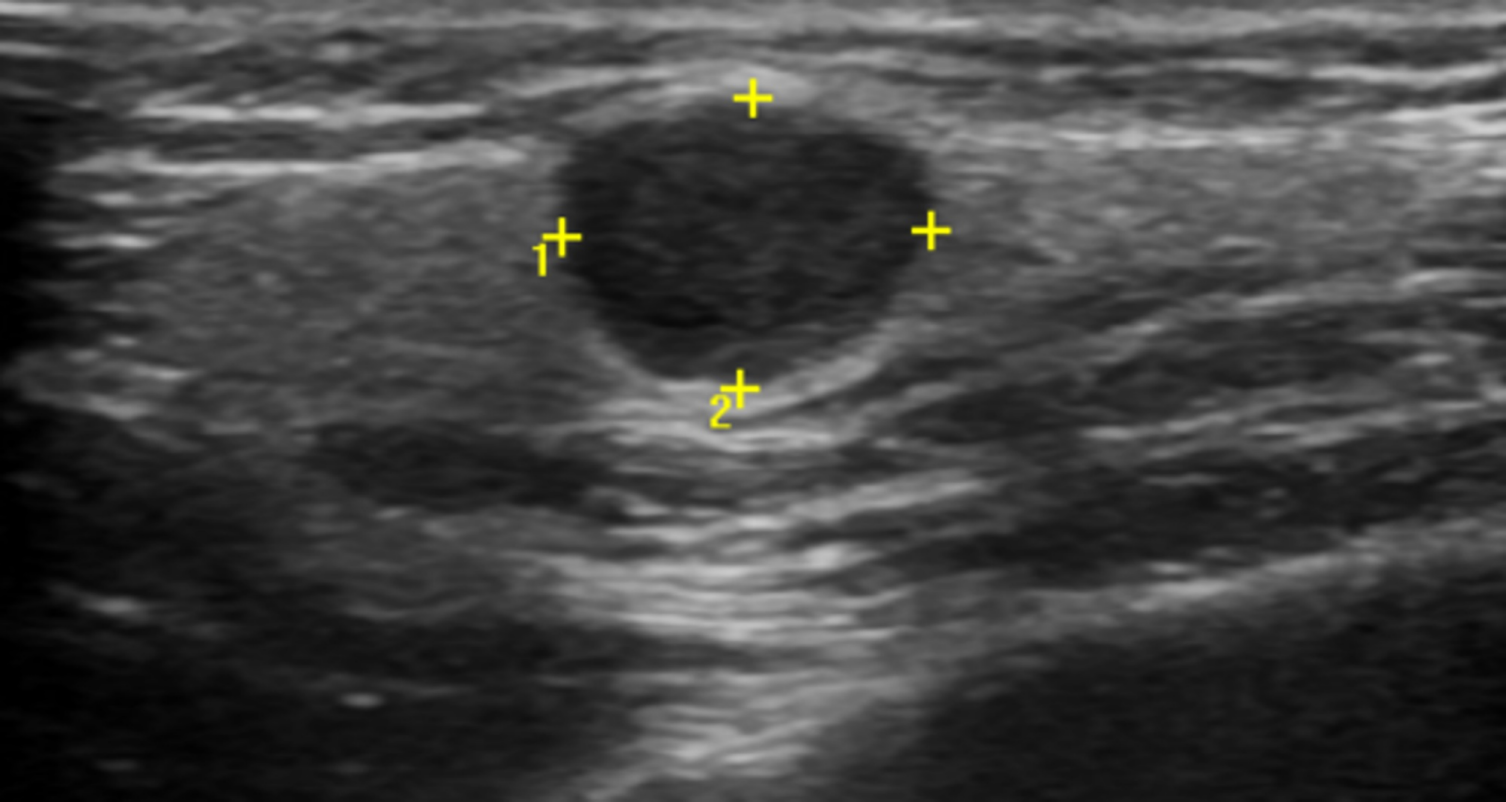
Ultrasonography showing the tumor in front of parotid gland above the masseter muscle

The rest of the examination revealed no pathologic alterations in the head and neck area. Ultrasonography showed a round tumor under the skin, outside of the buccinator muscle and around the anterior edge of the masseter muscle. Furthermore, this tumor was separated from the main parotid gland (Figure 2).

**Figure 3:**
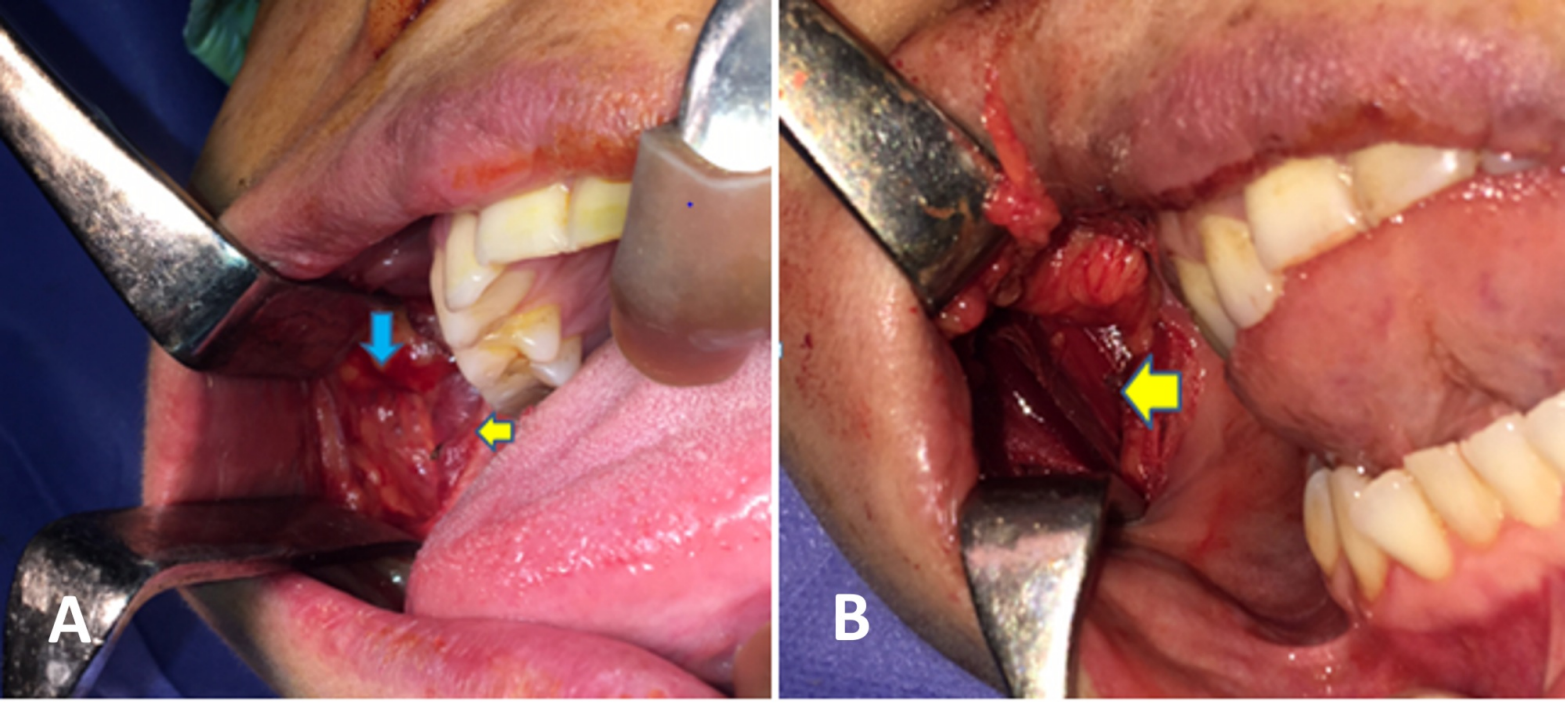
A, B intraoperative images showing masseter muscle (yellow arrow) and tumor (blue arrow)

**Figure 4:**
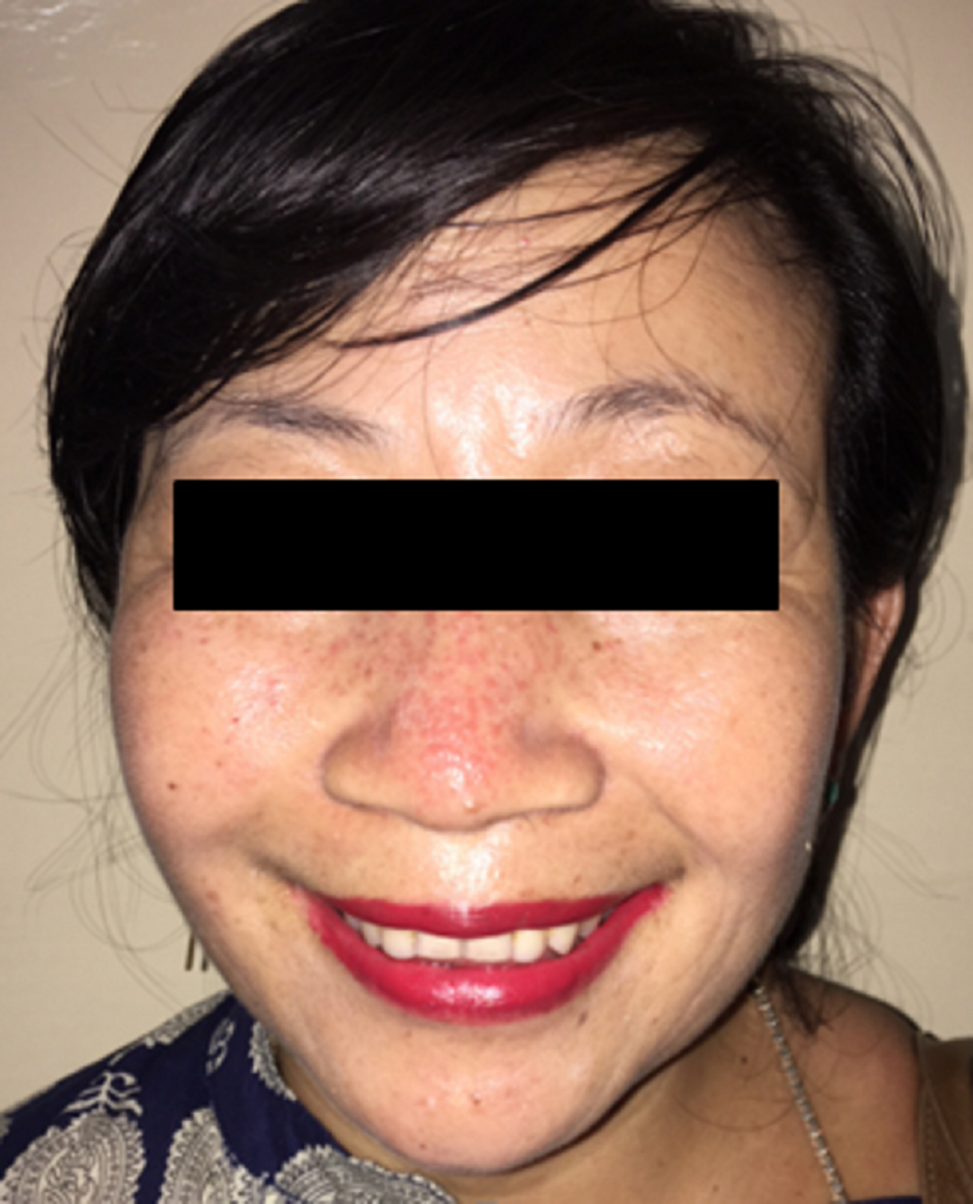
Patient’s face at the eight-month postoperative follow-up evaluation

The patient underwent fine-needle aspiration cytology and was diagnosed with a pleomorphic adenoma originating from an accessory parotid gland. After we discussed the risks of the procedure and the possibility of avoiding a scar with the patient, she provided informed consent to undergo surgery via a transoral approach. Transoral excision of the tumor was performed with assistance from an endoscopy for improved visualization (Figure 3). Frozen section was not performed in view of preoperative benign cytology report as well as no perioperative clinical suspicion. No intraoperative complications were encountered such as excessive bleeding. Postoperatively, the patient had minimal parotid swelling which was treated conservatively. Post surgery histopathology showed a benign pleomorphic adenoma. We monitored the patient via follow-up for eight months and found no postoperative complications, including pain, facial or auricular nerve weakness, salivary fistula, infection, tumor recurrence, Frey’s syndrome or depression deformity (Figure 4).

## Discussion

Tumors of accessory parotid glands are very rare, accounting for only 1% to 7.7% of all parotid gland tumors. Accessory parotid gland neoplasms should be suspected in any patient presenting with a mid cheek swelling [5-6]. Spiro et al. reported the occurrence of malignancy is >50% among accessory parotid gland tumors [7].

The preferred treatment of tumors arising from the accessory parotid gland is surgical resection either by traditional superficial parotidectomy or direct approaches. Preoperative fine-needle aspirate provides a valuable tool in the diagnosis and vital information for the appropriate surgical approach [8]. Fortunately, our patient had a benign lesion in preoperative cytology, it helped us in choosing the transoral approach than traditional open approach. Conventional parotidectomy approaches require a large flap elevation and leave an external scar with various postoperative complications like facial or auricular nerve weakness, salivary fistula, infection, tumor recurrence, Frey’s syndrome or depression deformity [4,9].

The direct transcutaneous approach leaves a scar on the face, which is highly visible and leaves depressed areas resulting from the excision. This approach is associated with a higher incidence (40%) of facial nerve branch damage because the buccal and zygomatic branches of the facial nerve are located superficial to accessory parotid gland tumors [7-8].

Given current technical developments in instrumentation and nerve monitoring systems, a transoral approach should be considered as an alternative to traditional approaches. The endoscopic technique has several benefits including reduced tissue damage, improved cosmesis, and fewer wound-related complications. Because of these benefits, head and neck surgeons have been performing endoscopic surgery by creating a working space in thyroidectomy, parathyroidectomy, submandibular gland dissection, thyroglossal duct cysts, and dermoid cysts, among others [10]. The availability of nerve monitoring systems may reduce the chances to injure the facial nerve which is the most severe complication in the transoral approach.

Oncological safety presents another challenge for using the transoral approach. McGurk et al. showed that extracapsular dissection as a viable alternative surgical approach to superficial parotidectomy in benign lumps, and the recurrence rate is almost similar without much postoperative morbidity. The potential risk in extracapsular dissection is encountering a malignant tumor masquerading as a benign lump. This can be mitigated by using intraoperative frozen section histological examinations [11-12]. Transoral surgery is not indicated for large and/or malignant tumors.

Endoscopic surgery is suitable for places proximal to natural cavities. Therefore, successful transoral surgery relies heavily on the careful selection of patients and tumor types. The availability of endoscopes, nerve monitoring systems, and frozen section analysis will likely increase the feasibility of transoral surgery in the future.

## Conclusions

Transoral parotid surgery is still in its infancy, and only a few case reports have been reported in the literature. This approach can be used safely for benign tumors of the accessory parotid gland in selected cases, especially when assisted with endoscopic and nerve monitoring systems. Intraoperative frozen section evaluations may help in diagnosing suspected malignant tumors.

## References

[1] Lin DT, Coppit GL, Burkey BB, Netterville JL (2004). Tumors of the accessory lobe of the parotid gland: a 10-year experience. Laryngoscope.

[2] Zhang DM, Wang YY, Liang QX, Song F, Chen WL, Zhang B (2015). Endoscopic-assisted resection of benign tumors of the accessory parotid gland. J Oral Maxillofac Surg.

[3] Langdon J (2002). The significance of the tumour capsule in pleomorphic adenoma: the changing face of conventional principles. Controversies in the Management of Salivary Gland Disease.

[4] Woo SH, Kim JP, Baek CH (2016). Endoscope-assisted extracapsular dissection of benign parotid tumors using hairline incision. Head Neck.

[5] Woo SH (2016). Endoscope-assisted transoral accessory parotid mass excision. Head Neck.

[6] Schmutzhard J, Schwentner IM, Andrle J, Gunkel AR, Sprinzl GM (2007). Resection of accessory parotid gland tumors through a peroral approach with facial nerve monitoring. J Craniofac Surg.

[7] Johnson FE, Spiro RH (1979). Tumors arising in accessory parotid tissue. Am J Surg.

[8] Choi HJ, Lee YM, Kim JH, Tark MS, Lee JH (2012). Wide excision of accessory parotid gland with anterior approach. J Craniofac Surg.

[9] Sun G, Hu Q, Tang E, Yang X, Huang X (2009). Diagnosis and treatment of accessory parotid-gland tumors. J Oral Maxillofac Surg.

[10] Arens C (2012). Transoral treatment strategies for head and neck tumors. Curr Top Otorhinolaryngol Head Neck Surg.

[11] McGurk M, Thomas BL, Renehan AG (2003). Extracapsular dissection for clinically benign parotid lumps: reduced morbidity without oncological compromise. Br J Cancer.

[12] Klintworth N, Zenk J, Koch M, Iro H (2010). Postoperative complications after extracapsular dissection of benign parotid lesions with particular reference to facial nerve function. Laryngoscope.

